# Outcome of contemporary unprotected left main percutaneous coronary intervention in patients with acute myocardial infarction

**DOI:** 10.3389/fcvm.2025.1682741

**Published:** 2026-01-09

**Authors:** Hong Nyun Kim, Jang Hoon Lee, Bo Eun Park, Yoon Jung Park, Jong Sung Park, Nam Kyun Kim, Youngjun Wi, Dong Heon Yang, Hun Sik Park, Yongkeun Cho, Myung Ho Jeong, Jong-Seon Park

**Affiliations:** 1 Department of Internal Medicine, Kyungpook National University Hospital, Daegu, Republic of Korea; 2 School of Medicine, Kyungpook National University, Daegu, Republic of Korea; 3 Department of Internal Medicine, Gwangju Veterans Hospital, Gwangju, Republic of Korea; 4 Department of Internal Medicine, Yeungnam University Hospital, Daegu, Republic of Korea

**Keywords:** acute myocardial infarction, unprotected left main coronary artery, percutaneous coronary intervention, complete revascularization, prognosis

## Abstract

**Background:**

Limited data are available on clinical characteristics and outcomes in patients with culprit or non-culprit left main coronary artery (LMCA) stenosis between ST-segment elevation myocardial infarction (STEMI) and non-STEMI.

**Methods:**

This study aimed to compare treatment pattern and outcome between STEMI and non-STEMI according to culprit and non-culprit LMCA stenosis. We examined 572 patients with LMCA stenosis from the Korean Acute Myocardial Infarction Registry–National Institute of Health database. Major adverse cardiac and cerebrovascular events (MACCE) were defined as all-cause death, nonfatal myocardial infarction (MI), repeat revascularization, cerebrovascular accident, rehospitalizations, and stent thrombosis.

**Results:**

In patients with culprit LMCA stenosis, cardiogenic shock (50.5% vs. 12.1%; *P* < 0.001) and use of mechanical hemodynamic support (48.5% vs. 11.0%; *P* < 0.001) were significantly greater in STEMI than in non-STEMI. In-hospital mortality (32.3% vs. 8.1%, *P* < 0.001) and 3-year MACCE (56.6% vs. 42.2%; log-rank *P* = 0.003) were significantly higher in STEMI. Intravascular ultrasound improved outcomes of culprit LMCA stenosis (23.1% vs. 68.1%, log-rank *P* = 0.001). Acute kidney injury, multiple organ failure, and cardiopulmonary resuscitation were independently associated with MACCE in STEMI. In patients with non-culprit LMCA stenosis, there were no significant differences in MACCE between STEMI and non-STEMI (31.3% vs. 34.8%, log-rank *P* = 0.530). Concurrent percutaneous coronary intervention (PCI) for non-culprit LMCA stenosis during PCI for other culprit vessel segments did not improve MACCE in STEMI (29.5% vs. 32.9%; log-rank *P* = 0.660).

**Conclusions:**

PCI for culprit LMCA stenosis is challenging in both STEMI and non-STEMI despite appropriate mechanical hemodynamic support. Concurrent PCI for non-culprit LMCA stenosis in STEMI does not improve MACCE.

## Graphical Abstract

**Figure:**
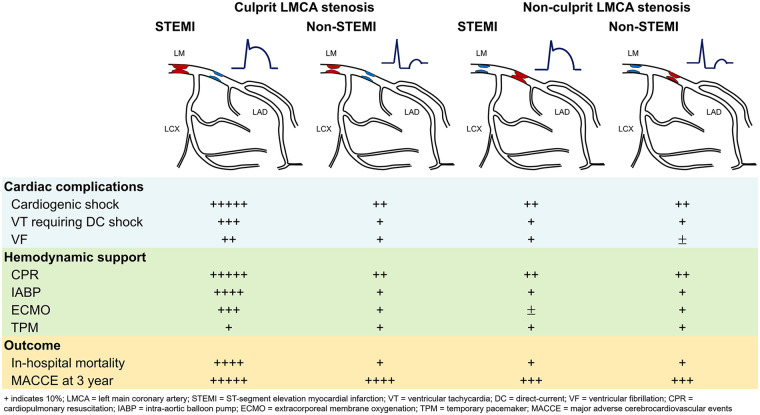

## Introduction

Advances in interventional techniques have improved the clinical outcome of percutaneous coronary intervention (PCI) of unprotected left main coronary artery (LMCA) stenosis compared with coronary artery bypass graft surgery (CABG) (1–5). Therefore, the number of PCI for LMCA stenosis has significantly increased over time. Recently, the guideline for PCI of LMCA has been upgraded from class III to class I in low anatomical complexity or class IIa in intermediate anatomical complexity in the European Society of Cardiology based on the results from a recent randomized controlled trial and meta-analysis (6–8). This indicates that the current guideline regarded PCI as an appropriate alternative to CABG in LMCA stenosis with low to intermediate anatomical complexity. However, in the context of acute myocardial infarction (AMI), PCI for LMCA stenosis is still associated with higher mortality and morbidity rates. Although the benefit of intravascular ultrasound (IVUS) examination has been proven in LMCA stenosis, the use of IVUS is not always possible in hemodynamically unstable patients with ST-elevation myocardial infarction (STEMI) or non-STEMI. Moreover, limited data are available about the outcome of concurrent PCI for non-culprit LMCA stenosis at the time of PCI for other culprit vessel segments. Therefore, we aimed to compare treatment pattern and outcome between STEMI and non-STEMI according to culprit and non-culprit LMCA stenosis.

## Methods

### Study design and patient population

The Korean Acute Myocardial Infarction Registry (KAMIR) is prospective, open, observational, multicenter, online registry of Korean patients with AMI supported by the National Institute of Health (NIH) since November 2011. AMI was diagnosed on the presence of acute myocardial injury detected by abnormal cardiac biomarkers in the setting of evidence of acute myocardial ischemia (9). Other details about KAMIR have been published (10).

All data about patients and procedural details were collected at the time of admission and followed prospectively at each hospital. Data were recorded on a web page-based report form with electronic encryption in the NIH database. This research was supported by a fund (2013-E63005-02) by Research of Korea Centers for Disease Control and Prevention. The protocol was approved by the ethics committee of each participating institution, and all patients gave written informed consent to participate in the study.

**Figure 1:**
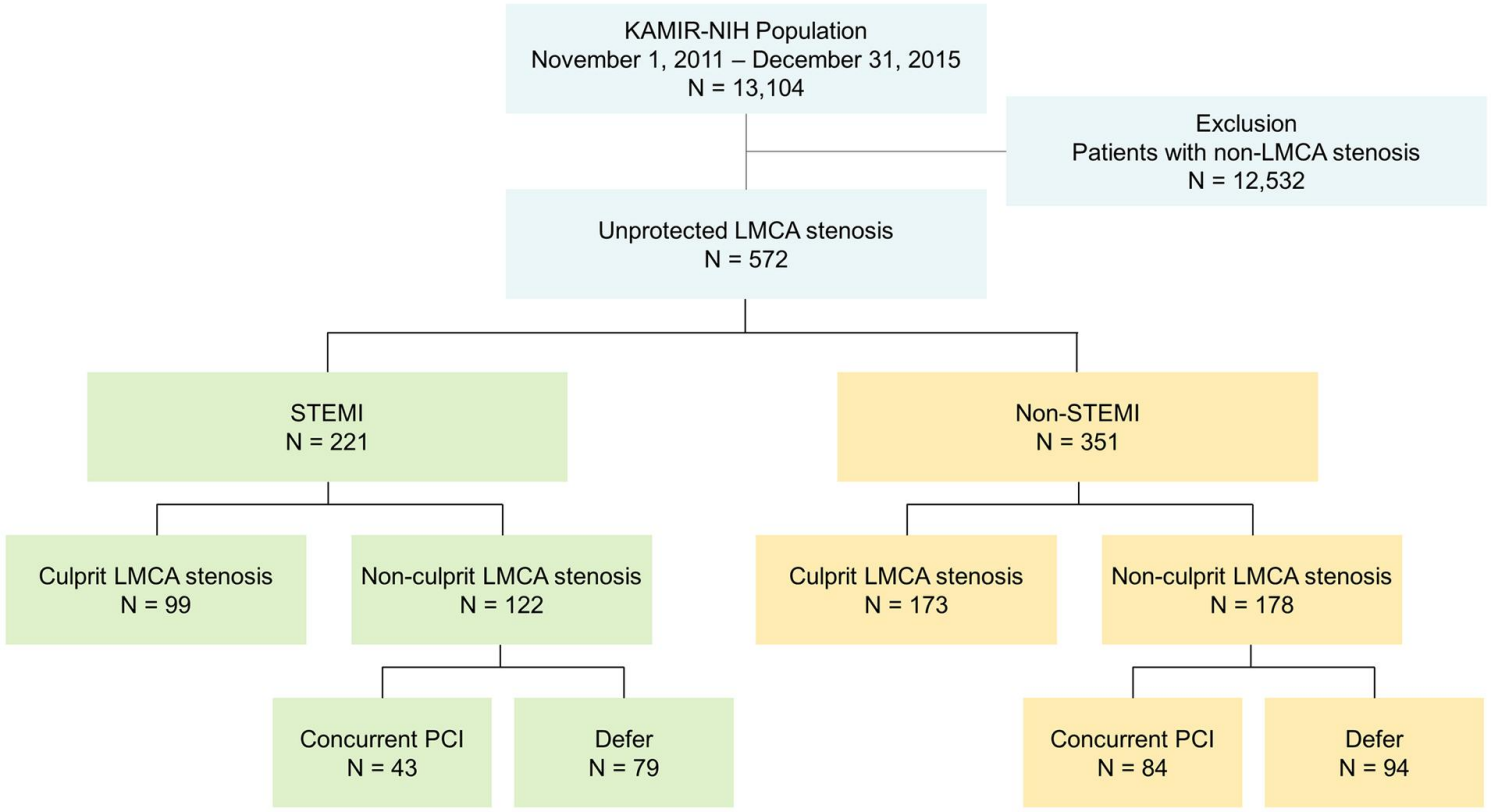
Flow diagram of the study subjects. KAMIR-NIH, Korea Acute Myocardial Infarction Registry-National Institutes of Health registry; LMCA, left main coronary artery; STEMI, ST-segment elevation myocardial infarction; PCI, percutaneous coronary intervention.

Between November 2011 and December 2015, 13,104 patients (9,686 men; mean age = 64.0 ± 12.6 years old) from 20 PCI centers who were diagnosed with AMI at admission were recruited (Figure 1). Among them, 572 patients with unprotected LMCA stenosis who underwent coronary angiography were finally analyzed in this study. Conceptual categorization of LMCA stenosis is shown in Supplementary Figure S1. A culprit lesion was defined as the lesion involved in the initial AMI, and a non-culprit lesion as any lesion in the entire coronary tree outside the culprit lesion. Culprit lesion was identified based on the findings by a coronary angiography as well as an electrocardiogram and transthoracic echocardiogram. Non-culprit lesion was defined as any lesion with ≥ 50% angiographic stenosis or a fractional flow reserve ≤0.80 in a ≥2.5 mm vessel. In patients with STEMI (*n* = 221), PCI for LMCA stenosis during index procedure was performed in 142 patients including 99 (44.8%) culprit LMCA stenoses and 43 (19.5%) non-culprit LMCA stenoses. In patients with non-STEMI (*n* = 351), PCI for LMCA stenosis during index procedure was performed in 257 non-STEMI patients including 173 (49.3%) culprit LMCA stenoses and 84 (23.9%) non-culprit LMCA stenoses. PCI was deferred in 79 (35.7%) non-culprit LMCA stenoses in STEMI and 94 (26.8%) in non-STEMI.

All procedures were performed with standard interventional techniques. Access site selection before the diagnostic or therapeutic procedure was at the discretion of the treating physician. Antiplatelet therapy and periprocedural anticoagulation followed the standard regimen. Before the procedure, all patients received a loading dose of aspirin (300 mg) and clopidogrel (300 mg or 600 mg) or prasugrel (60 mg) or ticagrelor (180 mg) at the discretion of the attending physician. In the catheterization laboratory, anticoagulation with a bolus of unfractionated heparin (75–100 U/kg) was administered to achieve an activated clotting time of >300 s. During the procedure, the use of IVUS and/or hemodynamic support such as intra-aortic balloon pump, extracorporeal membrane oxygenation, and cardiopulmonary resuscitation was at the operator's discretion. Routine use of post-procedure unfractionated heparin was not recommended except for patients requiring an intra-aortic balloon pump and/or extracorporeal membrane oxygenation. Glycoprotein IIb/IIIa receptor inhibitor was left to the discretion of the attending interventional cardiologist. After the procedure, use of guideline-directed medical therapy was mandatory, and the duration of dual antiplatelet agents was at the operator's discretion.

### Clinical outcomes

Major adverse cardiac and cerebrovascular events (MACCE) at 3 years were defined as all-cause death, nonfatal MI, repeat revascularization including repeated PCI and CABG, cerebrovascular accident including both ischemic and hemorrhagic stroke, rehospitalizations defined as all-cause readmissions without restriction to diagnostic category, and stent thrombosis. During the follow-up period, clinical outcome data were obtained by reviewing medical records and interviewing patients by telephone.

### Statistical analyses

Data were expressed as mean ± standard deviation for continuous variables and as percentages for categorical variables. Comparisons between baseline variables were assessed using Student's t-test for continuous variables and Pearson's chi-squared test for categorical variables. Patients were categorized into culprit LMCA stenosis and non-culprit LMCA stenosis. Baseline characteristics, angiographic and procedural findings, and outcomes were compared between STEMI and non-STEMI according to culprit LMCA stenosis and non-culprit LMCA stenosis. To determine predictors of MACCE, Cox proportional-hazards regression models were used to provide adjusted hazard ratios (HRs) with 95% confidence intervals (CIs). Variables with *P* < 0.05 in univariate analyses such as baseline characteristics including age > 70-year-old, male sex, Killip class > 1, hypertension, complications including atrial fibrillation, acute kidney injury, multi-organ failure, cardiogenic shock, ventricular tachycardia requiring cardioversion, and treatment of complications including cardiopulmonary resuscitation, intra-aortic balloon pump, and extracorporeal membrane oxygenation were included in multivariate analysis model. MACCE were compared using Kaplan–Meier survival curves. For all analyses, a two-sided *P* < 0.05 was considered statistically significant. Statistical analysis was performed using IBM SPSS statics (version 30.0; IBM Corp., Armonk, NY, USA) and R software (version 4.5.2; R Foundation for Statistical Computing, Vienna, Austria).

### Ethics statement

This study protocol was reviewed and approved by the Institutional Review Board of Kyungpook National University School of Medicine (approval No. 2022-01-011) and informed consent was submitted by all patients when they were enrolled.

## Results

Baseline characteristics are presented in Table 1. Overall, mean age was 67.0 ± 11.6 years old and 448 patients were men. In patients with culprit LMCA stenosis, patients with STEMI had significantly lower systolic blood pressure (*P* < 0.001), higher Killip class (*P* < 0.001), higher prevalence of previous angina (*P* = 0.015), and lower left ventricular ejection fraction (*P* < 0.001). The use of beta blockers (*P* = 0.002), angiotensin-converting enzyme inhibitors/angiotensin II receptor blockers (*P* < 0.001), and statin (*P* < 0.001) was significantly lower in STEMI compared with non-STEMI. In patients with non-culprit LMCA stenosis, patients with STEMI had significantly lower systolic blood pressure (*P* = 0.001). The use of clopidogrel (*P* = 0.017) was lower while the use of ticagrelor (*P* < 0.001) was greater in STEMI compared with non-STEMI. There was no significant difference in use of dual-antiplatelet therapy between STEMI and non-STEMI in both culprit (*P* = 0.671) and non-culprit LMCA stenosis (*P* = 0.569).

**Table 1:** Baseline characteristics of study subjects.

Variables	Culprit LMCA stenosis		*P* value	Non-culprit LMCA stenosis		*P* value
	STEMI (*n* = 99)	Non-STEMI (*n* = 173)		STEMI (*n* = 122)	Non-STEMI (*n* = 178)	
Demographics						
Age (year)	65.6 ± 12.4	67.0 ± 12.0	0.374	68.0 ± 11.8	67.1 ± 10.6	0.538
Male	81 (81.8%)	130 (75.2%)	0.204	99 (81.1%)	138 (77.5%)	0.450
BMI (kg/m^2^)	23.5 ± 3.3	23.7 ± 3.3	0.638	24.2 ± 3.2	23.8 ± 3.1	0.414
Initial presentation						
Systolic BP (mmHg)	97.2 ± 38.8	127.9 ± 35.4	<0.001	120.6 ± 30.6	133.3 ± 32.6	0.001
Heart rate (beats/min)	83.3 ± 27.7	84.5 ± 21.4	0.713	78.1 ± 20.6	82.6 ± 20.6	0.061
Killip class >1	61 (61.6%)	56 (32.4%)	<0.001	30 (24.6%)	44 (24.7%)	0.980
Past medical history						
Hypertension	51 (51.5%)	93 (53.8%)	0.722	68 (55.7%)	104 (58.4%)	0.644
Diabetes mellitus	37 (37.4%)	63 (36.4%)	0.875	39 (32.0%)	69 (38.8%)	0.228
Hyperlipidemia	12 (12.1%)	21 (12.1%)	0.997	13 (10.7%)	24 (13.5%)	0.464
Current smoking	37 (37.4%)	49 (28.3%)	0.122	36 (29.5%)	57 (32.0%)	0.644
Previous MI	11 (11.1%)	12 (6.9%)	0.234	15 (12.3%)	19 (10.7%)	0.664
Previous angina	11 (11.3%)	40 (23.1%)	0.015	13 (10.7%)	29 (16.3%)	0.167
LVEF by volume (%)	40.5 ± 12.0	51.6 ± 13.8	<0.001	48.6 ± 11.4	50.3 ± 12.6	0.260
Medical therapy						
Aspirin	96 (97.0%)	171 (98.8%)	0.268	120 (98.4%)	178 (100.0%)	0.087
Clopidogrel	75 (75.8%)	147 (85.0%)	0.059	96 (78.7%)	158 (88.8%)	0.017
Prasugrel	13 (13.1%)	17 (9.8%)	0.403	14 (11.5%)	17 (9.6%)	0.591
Ticagrelor	21 (21.2%)	36 (20.8%)	0.937	39 (32.0%)	24 (13.5%)	<0.001
Dual-Antiplatelet	96 (97.0%)	170 (98.3%)	0.671	120 (98.4%)	177 (99.4%)	0.569
Beta-blockers	58 (58.6%)	133 (76.9%)	0.002	97 (79.5%)	143 (80.3%)	0.860
ACE-Is/ARBs	51 (51.5%)	130 (75.1%)	<0.001	87 (71.3%)	131 (73.6%)	0.663
Statins	64 (64.6%)	150 (86.7%)	<0.001	107 (87.7%)	162 (91.0%)	0.355

Data expressed as mean ± SD or number (percent).

LMCA, left main coronary artery; STEMI, ST-segment elevation myocardial infarction; BMI, body mass index; BP, blood pressure; MI, myocardial infarction; LVEF, left ventricular ejection fraction; ACE-Is, angiotensin converting enzyme inhibitors; ARB, angiotensin II receptor blockers.

**Figure 2:**
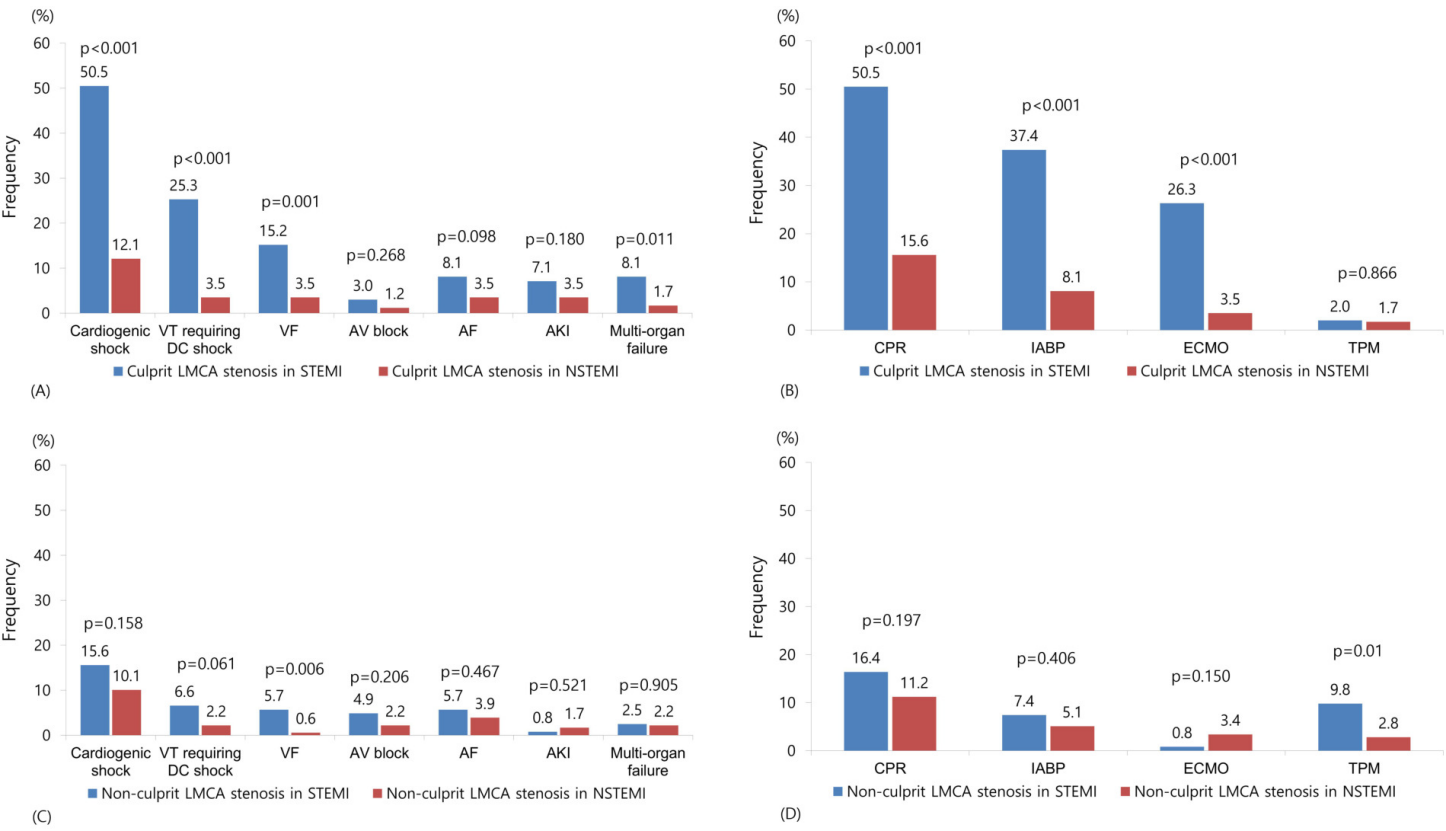
Frequency of complications and hemodynamic support in patients with culprit **(A,B)** or non-culprit **(C,D)** LMCA stenosis. VT, ventricular tachycardia; DC, direct current; VF, ventricular fibrillation; AV, atrioventricular; AF, atrial fibrillation; AK, acute kidney injury; LMCA, left main coronary artery; CPR, cardiopulmonary resuscitation; IABP, intra-aortic balloon pump; ECMO, extracorporeal membrane oxygenation; TPM, temporary pacemaker.

Angiographic and procedural characteristics are summarized in Table 2. In patients with culprit LMCA stenosis, patients with STEMI had significantly lower pre TIMI 0 flow (*P* < 0.001) and lower post TIMI 3 flow (*P* = 0.012). IVUS (*P* < 0.001) was less frequently used in STEMI compared with non-STEMI. Among complications, cardiogenic shock (*P* < 0.001), ventricular tachycardia requiring cardioversion (*P* < 0.001), ventricular fibrillation (*P* = 0.001), and multi-organ failure (*P* = 0.011) were more frequent in STEMI (Figure 2A). Cardiopulmonary resuscitation (*P* < 0.001), intra-aortic balloon pump (*P* < 0.001), and extracorporeal membrane oxygenation (*P* < 0.001) were more frequently used in STEMI (Figure 2B). In patients with non-culprit LMCA stenosis, patients with STEMI had significantly lower pre TIMI 0 flow (*P* < 0.001) and lower post TIMI 3 flow (*P* = 0.012). The total number of stents (*P* = 0.049) and the use of IVUS (*P* < 0.001) was lower in STEMI compared with non-STEMI. Among complications, ventricular fibrillation (*P* = 0.006) was more frequent in STEMI (Figure 2C). Temporary pacemaker (*P* = 0.01) was more frequently used in STEMI (Figure 2D).

**Table 2:** Angiographic characteristics of study subjects.

Variables	Culprit LMCA stenosis		*P* value	Non-culprit LMCA stenosis		*P* value
	STEMI (*n* = 99)	Non-STEMI (*n* = 173)		STEMI (*n* = 122)	Non-STEMI (*n* = 178)	
No. of diseased vessel						
LM isolated (%)	22 (22.2%)	33 (19.1%)	0.534	0 (0.0)	0 (0.0)	>0.999
LM complex (%)	77 (77.8%)	140 (80.9%)	0.534	122 (100.0%)	178 (100.0%)	>0.999
Lesion type			0.217			0.378
Type A (%)	2 (2.0%)	7 (4.0%)		1 (0.8%)	4 (2.2%)	
Type B1 (%)	9 (9.1%)	18 (10.4%)		9 (7.4%)	19 (10.7%)	
Type B2 (%)	42 (42.4%)	89 (51.4%)		32 (26.2%)	54 (30.3%)	
Type C (%)	46 (46.5%)	59 (34.1%)		80 (65.6%)	101 (56.7%)	
Pre TIMI 0 (%)	61 (61.6%)	163 (94.2%)	<0.001	53 (43.4%)	131 (73.6%)	<0.001
Post TIMI 3 (%)	90 (90.9%)	169 (97.7%)	0.012	111 (91.0%)	172 (96.6%)	0.038
Stent type			0.257			0.064
BMS (%)	1 (1.1%)	1 (0.6%)		1 (0.9%)	4 (2.4%)	
EES (%)	47 (50.5%)	79 (48.2%)		71 (64.0%)	84 (51.2%)	
ZES (%)	19 (20.4%)	49 (29.9%)		15 (13.5%)	45 (27.4%)	
BES (%)	11 (11.8%)	21 (12.8%)		17 (15.3%)	22 (13.4%)	
Other DES (%)	15 (16.1%)	14 (8.5%)		7 (6.3%)	9 (5.5%)	
Stent no.	1.72 ± 0.93	1.86 ± 1.08	0.290	1.80 ± 1.16	2.09 ± 1.33	0.049
Stent length (mm)	23.5 ± 14.9	22.9 ± 13.4	0.722	31.7 ± 19.0	31.1 ± 19.2	0.790
IVUS (%)	26 (26.5%)	88 (52.4%)	<0.001	38 (32.2%)	79 (47.0%)	0.012

Data expressed as mean ± SD or number (percent).

LMCA, left main coronary artery; STEMI, ST-segment elevation myocardial infarction; LM, left main; BMS, bare-metal stent; EES, everolimus eluting stent; ZES, zotarolumus eluting stent; BES, biolimus eluting stent; DES, drug-eluting stent; IVUS, intravascular ultrasound.

**Figure 3:**
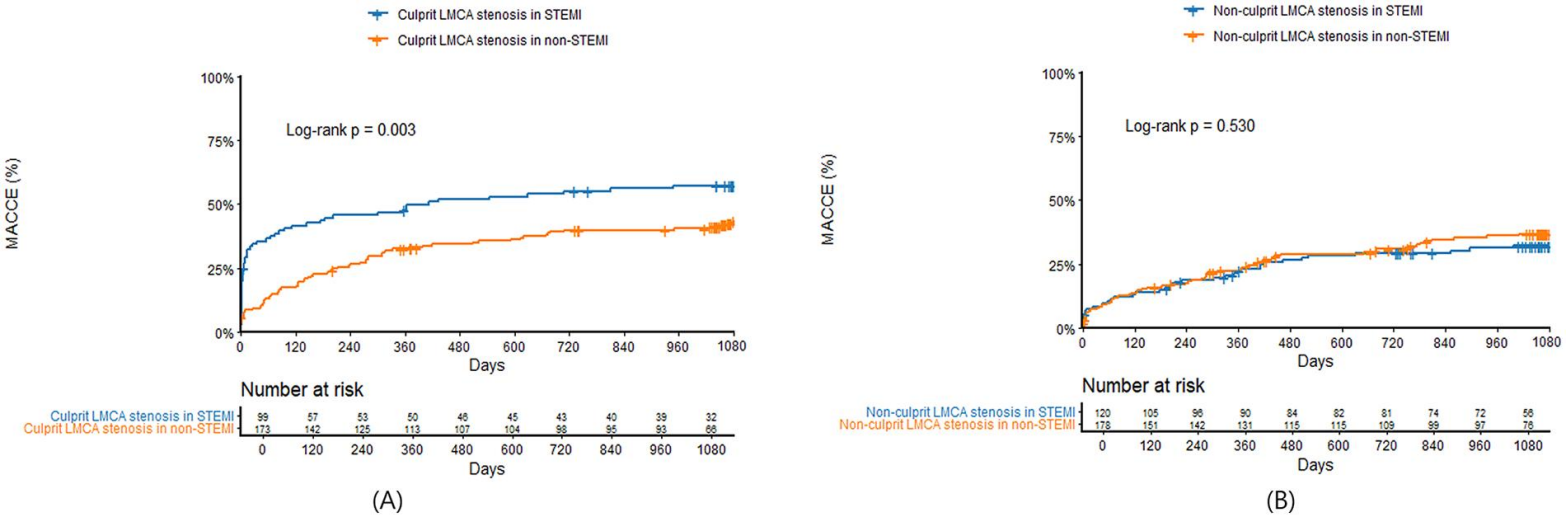
Kaplan–Meier survival curves comparing 3-year major adverse cardiac and cerebrovascular events between STEMI and non-STEMI in patients with culprit **(A)** or non-culprit **(B)** LMCA stenosis. LMCA, left main coronary artery; STEMI, ST-segment elevation myocardial infarction; MACCE, major adverse cardiac and cerebrovascular events; PCI, percutaneous coronary intervention.

**Figure 4:**
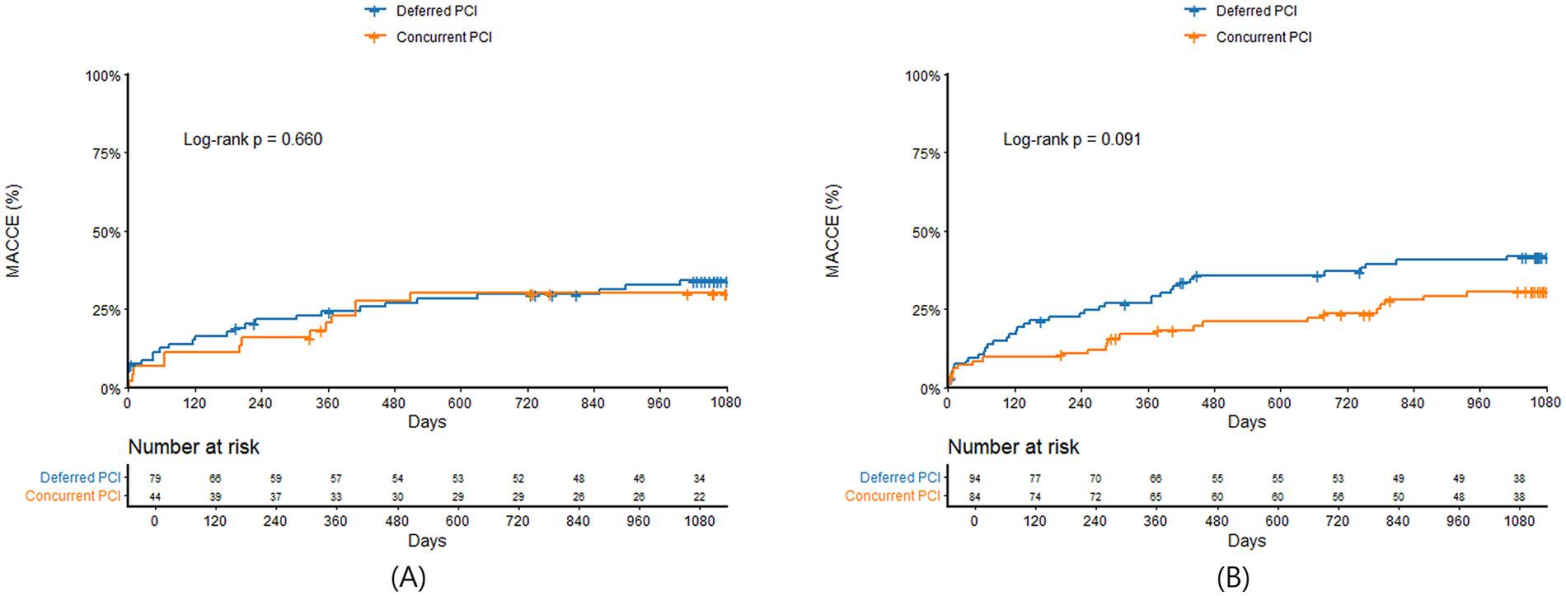
Kaplan–Meier survival curves comparing 3-year major adverse cardiac and cerebrovascular events between concurrent PCI and deferred PCI for non-culprit LMCA stenosis in STEMI **(A)** and non-STEMI **(B)** LMCA, left main coronary artery; STEMI, ST-segment elevation myocardial infarction; MACCE, major adverse cardiac and cerebrovascular events; PCI, percutaneous coronary intervention.

Clinical outcomes were presented in Table 3. In patients with culprit LMCA stenosis, in-hospital mortality was significantly higher in STEMI compared with non-STEMI (32.3% vs. 8.1%, *P* < 0.001). In landmark analysis at the 30-day time point, MACCE before 30 days were significantly higher in STEMI patients with culprit LMCA stenosis compared with non-STEMI (34.3% vs. 9.2%, log-rank *P* < 0.001), but there was no significant difference between the two groups after 30 days (25.0% vs. 30.1%, log-rank *P* = 0.455). MACCE at 3 years was significantly higher in STEMI compared with non-STEMI (56.6% vs. 42.2%, log-rank *P* = 0.003) (Figure 3A), which was mainly driven by all-cause mortality (43.4% vs. 24.9%, log-rank *P* < 0.001) and cardiac mortality (38.4% vs. 19.1%, log-rank *P* < 0.001). In patients with non-culprit LMCA stenosis, there were no significant differences in in-hospital mortality (7.4% vs. 7.3%, *P* = 0.981) and 3-year MACCE (31.3% vs. 34.8%, log-rank *P* = 0.530) (Figure 3B) between STEMI and non-STEMI. In subgroup analysis, patients were divided into concurrent PCI during index procedure and deferred PCI for non-culprit LMCA stenosis. Compared with deferred PCI for non-culprit LMCA stenosis, concurrent PCI during index procedure tended to be lower in 3-year MACCE in non-STEMI (28.6% vs. 40.4%; log-rank *P* = 0.091), but not in STEMI (29.5% vs. 32.9%; log-rank *P* = 0.660) (Figures 4A,B). There was no significant difference between Figures 4A,B (interaction *P* = 0.578).

**Table 3:** Clinical outcomes of study subjects.

Outcomes	Culprit LMCA stenosis		*P* value	Non-culprit LMCA stenosis		*P* value
	STEMI (*n* = 99)	Non-STEMI (*n* = 173)		STEMI (*n* = 122)	Non-STEMI (*n* = 178)	
Primary outcome						
MACCE	56 (56.6%)	73 (42.2%)	0.003	38 (31.3%)	62 (34.8%)	0.530
Secondary outcome						
Death	43 (43.4%)	43 (24.9%)	<0.001	17 (13.9%)	26 (14.6%)	0.580
Cardiac death	38 (38.4%)	33 (19.1%)	<0.001	13 (10.7%)	16 (9.0%)	0.997
Noncardiac death	5 (5.1%)	10 (5.8%)	0.790	4 (3.3%)	10 (5.6%)	0.347
Nonfatal MI	5 (5.1%)	13 (7.5%)	0.855	3 (2.5%)	11 (6.2%)	0.129
Revascularization	10 (10.1%)	22 (12.7%)	0.881	19 (15.6%)	34 (19.1%)	0.385
Repeat PCI	8 (8.1%)	19 (11.0%)	0.957	18 (14.8%)	29 (16.3%)	0.672
CABG	2 (2.0%)	3 (1.7%)	0.613	1 (0.8%)	5 (2.8%)	0.233
CVA	3 (3.0%)	1 (0.6%)	0.047	1 0.8%)	1 (0.6%)	0.813
Rehospitalization	6 (6.1%)	14 (8.1%)	0.881	6 (4.9%)	11 (6.2%)	0.624
Stent thrombosis	0 (0.0%)	1 (0.6%)	0.520	1 (0.8%)	1 (0.6%)	0.793

Data expressed as number (percent).

LMCA, left main coronary artery; STEMI, ST-segment elevation myocardial infarction; MACCE, major adverse cerebrocardiovascular event; MI, myocardial infarction; PCI, percutaneous coronary intervention; CABG, coronary artery bypass grafting; CVA, cerebrovascular accident.

Cardiac complications, hemodynamic support, and outcomes in patients with culprit or non-culprit LMCA stenosis are summarized in Graphic abstract. The culprit LMCA stenosis in STEMI was an independent predictor of 3-year MACCE (HR 1.83, 95% CI 1.27–2.63; *P* = 0.001), whereas the non-culprit LMCA stenosis in STEMI (HR 0.85, 95% CI 0.56–1.29; *P* = 0.462), concomitant PCI of non-culprit LMCA in STEMI (HR 1.03, 95% CI 0.52–2.07; *P* = 0.914) and non-STEMI (HR 0.66, 95% CI 0.39–1.10; *P* = 0.112) was not an independent predictor of 3-year MACCE after adjusting for confounding variables. In STEMI patients with culprit LMCA stenosis, acute kidney injury (HR 8.85, 95% CI 2.44–32.11; *P* = 0.001), multi-organ failure (HR 4.95, 95% CI 1.66–14.72; *P* = 0.004), and cardiopulmonary resuscitation (HR 2.24, 95% CI 1.02–4.90; *P* = 0.044) was an independent predictor of 3-year MACCE (Table 4). When IVUS was performed, 3-year MACCE was significantly lower in IVUS users compared with non-IVUS users (33.3% vs. 57.9%, log-rank *P* < 0.001), particularly in STEMI (23.1% vs. 68.1%, log-rank *P* < 0.001), but not in non-STEMI (36.4% vs. 48.8%, log-rank *P* = 0.078) (Supplementary Figure S2). IVUS use was an independent predictor of 3-year MACCE (HR 0.72, 95% CI 0.53–0.98; *P* = 0.041) after adjusting for confounding variables.

**Table 4:** Cox proportional-hazards model for 3-year major adverse cardiac and cerebrovascular events.

Variables	Hazard ratio	95% Confidence interval	*P* value
Baseline characteristics			
Age >70 year-old	1.87	0.87–3.98	0.105
Male	0.87	0.39–1.93	0.745
Killip class >1	1.73	0.83–3.59	0.139
Hypertension	1.49	0.78–2.84	0.218
Complications			
Atrial fibrillation	0.35	0.11–1.05	0.062
Acute kidney injury	8.85	2.44–32.1	0.001
Multi-organ failure	4.95	1.66–14.7	0.004
VT requiring DC cardioversion	0.93	0.45–1.93	0.855
Treatment of complications			
Cardiopulmonary resuscitation	2.24	1.02–4.90	0.044
IABP	0.89	0.41–1.92	0.767
ECMO	1.82	0.83–3.95	0.129

VT, ventricular tachycardia; DC, direct current; CPR, cardiopulmonary resuscitation; IABP, intra-aortic balloon pump; ECMO, extracorporeal membrane oxygenation.

## Discussion

The principal findings of this large observational study are as follows. First, although hemodynamic support devices were more frequently used in STEMI, challenging clinical scenarios and events were significantly greater for culprit LMCA stenosis in STEMI than non-STEMI. Second, IVUS improved outcome of culprit LMCA stenosis even in STEMI. Third, in patients with non-culprit LMCA stenosis, there was no significant difference in outcome between STEMI and non-STEMI. Fourth, concurrent PCI for non-culprit LMCA stenosis tended to improve outcome in non-STEMI, but not in STEMI, although this trend was not statistically significant.

To the best of our knowledge, there were only few studies comparing clinical characteristics and outcome of LMCA stenosis between STEMI and non-STEMI according to culprit and non-culprit lesion. Although emergent PCI for LMCA stenosis is still challenging even for an experienced interventional cardiologist, limited data are available on these challenging clinical scenarios. Our study provides comprehensive data for current status of PCI for culprit or non-culprit LMCA stenosis in STEMI and non-STEMI. The most intriguing finding of this study is that clinical events of LMCA stenosis in both STEMI and non-STEMI are significantly higher than those of previously published studies. Previous studies reported in-hospital mortality of about 10% in STEMI and 6%–7% in non-STEMI treated with LMCA stenosis (11–14), compared to 32.3% in-hospital mortality in STEMI and 8.1% in-hospital mortality in non-STEMI treated as culprit LMCA stenosis in our study.

There are several plausible explanations why the mortality rate in our study is higher than previous studies and why our STEMI patients have higher mortality than non-STEMI patients with culprit LMCA stenosis. First, the prevalence of hemodynamically unstable patients is higher in our study. In previous studies, only 8%–12% of STEMI patients who underwent primary PCI for culprit LMCA stenosis had cardiogenic shock or cardiac arrest (11–13). However, cardiogenic shock and cardiopulmonary resuscitation were observed in 50% of our STEMI patients with culprit LMCA stenosis. In addition, cardiogenic shock and life-threatening ventricular arrhythmia was more frequently observed in STEMI compared with non-STEMI. Previous studies reported a 66% prevalence of cardiogenic shock and a 61% in-hospital mortality in patients treated with emergency PCI for LMCA stenosis (15, 16). Furthermore, the use of optimal medical therapy, including beta-blockers, angiotensin-converting enzyme inhibitors or angiotensin II receptor blockers, and statins, was significantly lower in patients with STEMI than in those with non-STEMI who had culprit LMCA stenosis, which is thought to be attributable to the higher risk of hemodynamic instability, potential contraindications, and the greater severity of clinical presentation. Optimal medical therapy is well known to improve the prognosis of patients with AMI. Therefore, the lower use of these medications likely contributed to the observed differences in clinical outcomes. These data are in line with our results and explain why our STEMI patients with LMCA stenosis have remarkably high in-hospital mortality and during the follow-up.

Second, severity of LMCA stenosis accompanied by concurrent lesions of other coronary segments may affect outcome. More than 80% of our patients with LMCA stenosis have various degrees of multivessel stenosis in other coronary segment, compared with 40%–60% in other previous reports. The prognosis of patients with LMCA stenosis with additional lesions on other vessel segments is worse than patients with isolated LMCA stenosis. This indicates that LMCA stenosis with widespread pattern of anatomical lesions has more ischemic burden and substrate for ventricular arrhythmia, hence the reason why our study showed a higher cardiac mortality rate than previous studies.

Third, patients with LMCA stenosis usually have a heterogeneous condition. Therefore, various degrees of developing in-hospital noncardiac complications may affect outcome. In our study, developing acute kidney injury and multi-organ failure during hospitalization was a major determinant of clinical outcomes. The prevalence of these complications was significantly higher in STEMI with culprit LMCA stenosis compared with non-STEMI patients. This is another reason why our STEMI patients have higher mortality than non-STEMI patients with culprit LMCA stenosis. Not surprisingly, in patients with non-culprit LMCA stenosis, no significant difference was found in outcome between STEMI and non-STEMI because there was no difference in the prevalence of noncardiac complications and cardiac complications between the two groups.

Another novel finding of our results is that our study provides important data regarding the role of IVUS and multivessel PCI on outcome in AMI patients with LMCA stenosis. First, our analysis supports the use of IVUS in PCI for hemodynamically stable patients with culprit LMCA stenosis even in AMI, as the current guideline recommends IVUS for assessing severity of stable LMCA stenosis (Class IIa) (6, 17–19). However, the role of IVUS in hemodynamically unstable patients remains controversial.

Second, our analysis does not support concurrent PCI of non-culprit LMCA stenosis during the primary PCI for other culprit vessel segments in STEMI. However, interestingly, multivessel PCI including non-culprit LMCA stenosis improved outcome in non-STEMI. The mechanisms of this dichotomy are somewhat unclear. Although other studies showed possible benefit with non-culprit vessel PCI in hemodynamically stable AMI patients (20–23), recent randomized controlled trial showed that multivessel PCI was not better than culprit-only PCI in patients with cardiogenic shock and AMI (24, 25). Therefore, it seems that the high prevalence of cardiogenic shock and ventricular arrhythmia in STEMI patients with non-culprit LMCA stenosis may be at least partially responsible. The current guideline also does not recommend routine PCI of non-culprit lesion during primary PCI in cardiogenic shock (class III).

Our research has several limitations to consider. First, since the KAMIR-NIH was an observational study, we cannot completely exclude the possibility of residual confounding factors. Therefore, our results should only be regarded as hypothesis generating. Second, the choice of PCI for LMCA stenosis was not randomized and left to the operator's best discretion. In addition, PCI strategy for non-culprit lesion and use of hemodynamic support devices were also left to the operator's discretion. Third, we were not able to control unmeasured factors such as individual operator experience, hospital resources, and regional practice variations for treatment choices which may affect outcomes. Therefore, differences in technical skills among each operator may cause bias; hence the results need to be interpreted with caution. Fourth, anatomic complexity such as bifurcation lesions, calcifications and so on may impact treatment decision and outcome. However, anatomic complexity was not controlled in our registry. Furthermore, previous studies have mainly demonstrated short-term outcomes (26), whereas our study provides updated data on 3-year MACCE and independent prognostic factors in STEMI patients with culprit LMCA stenosis. Therefore, the limitations should not undermine the strength of this study that includes overall patient encounters in day-to-day clinical practice.

In conclusion, PCI for culprit LMCA stenosis is still challenging for both STEMI and non-STEMI despite appropriate mechanical hemodynamic support. Clinical events remain high in both culprit and non-culprit LMCA stenosis in STEMI and non-STEMI. Concurrent PCI for non-culprit LMCA stenosis improves outcome in non-STEMI. However, further studies are required regarding the role of concurrent PCI for non-culprit LMCA stenosis during primary PCI for other culprit vessel segments.

## Data Availability

This study is based on data from The Korean Acute Myocardial Infarction Registry (KAMIR) (https://www.ksmi.re.kr/eng/). The datasets presented in this article are not readily available: the KAMIR database is managed by the central coordinating center, and access to the data is granted to researchers solely for research purposes upon formal request. Requests to access the datasets should be directed to corresponding author (Janghoon Lee, ljhmh75@knu.ac.kr).

## Supplementary material

Supplementary Figure S1 Conceptual categorization of left main coronary artery stenosis of this study. LMCA, left main coronary artery; STEMI, ST-segment elevation myocardial infarction.

Supplementary Figure S2 Kaplan–Meier survival curves for 3-year major adverse cardiac and cerebrovascular events between IVUS and non-IVUS use for culprit LMCA stenosis in overall **(A)**, STEMI **(B)**, and non-STEMI **(C)**. IVUS, intravascular ultrasound; LMCA, left main coronary artery; STEMI, ST-segment elevation myocardial infarction.
