# Supplementary figures and images for: Outcome of contemporary unprotected left main percutaneous coronary intervention in patients with acute myocardial infarction
Source: Front Cardiovasc Med. 2026 Jan 9;12:1682741. doi: 10.3389/fcvm.2025.1682741 (PMC12827615; doi:10.3389/fcvm.2025.1682741)

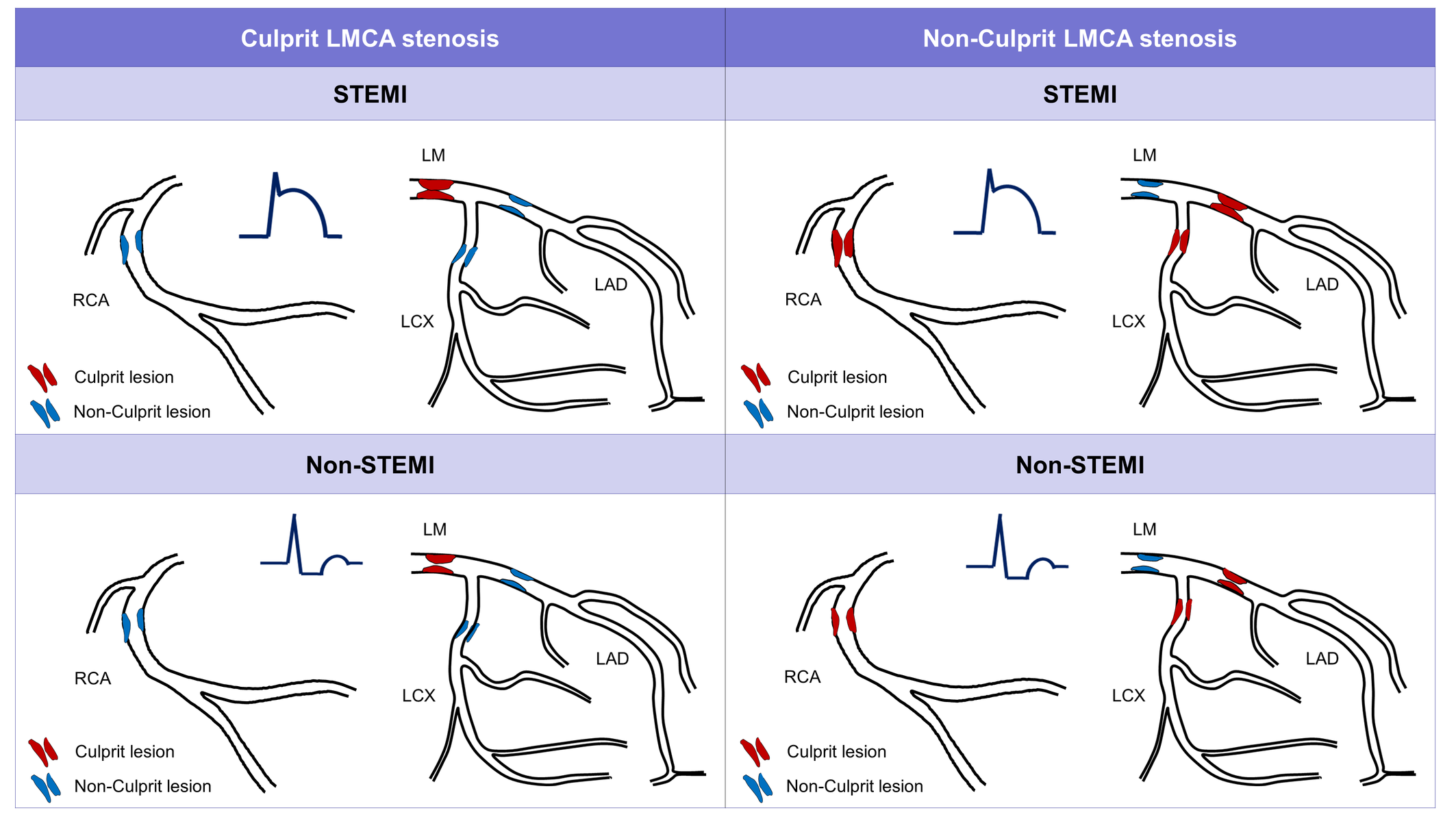

Supplement: Supplementary Figure S1 — Conceptual categorization of left main coronary artery stenosis of this study. LMCA, left main coronary artery; STEMI, ST-segment elevation myocardial infarction. [file Image1.tif]

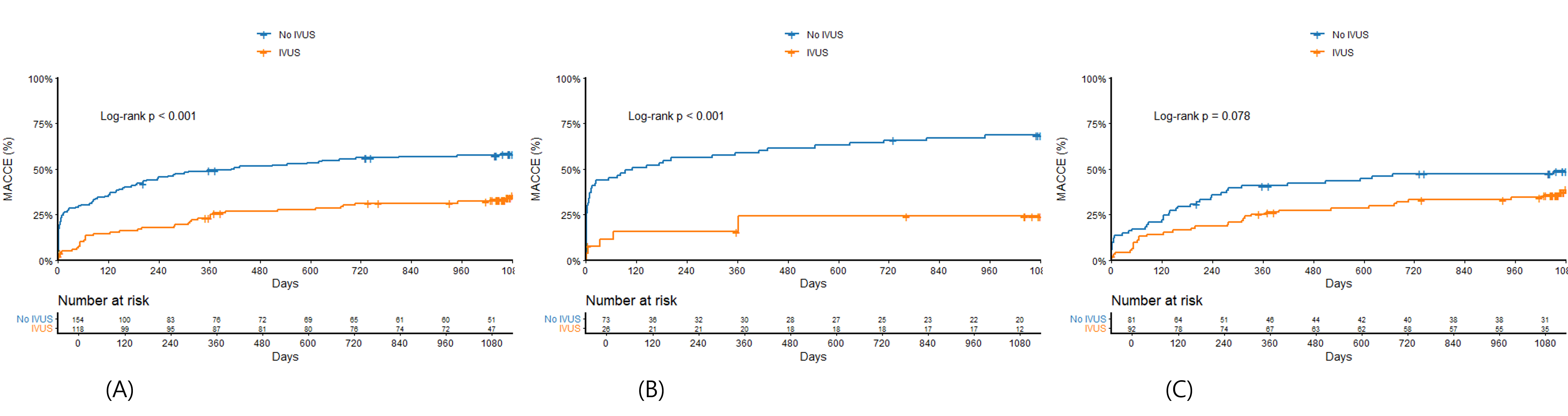

Supplement: Supplementary Figure S2 — Kaplan–Meier survival curves for 3-year major adverse cardiac and cerebrovascular events between IVUS and non-IVUS use for culprit LMCA stenosis in overall (A), STEMI (B), and non-STEMI (C). IVUS, intravascular ultrasound; LMCA, left main coronary artery; STEMI, ST-segment elevation myocardial infarction. [file Image2.tif]
